# Encephalitis caused by a Lyssavirus in fruit bats in Australia.

**DOI:** 10.3201/eid0204.960408

**Published:** 1996

**Authors:** G C Fraser, P T Hooper, R A Lunt, A R Gould, L J Gleeson, A D Hyatt, G M Russell, J A Kattenbelt

**Affiliations:** NSW Department of Agriculture, Wollongbar, Australia.

## Abstract

This report describes the first pathologic and immunohistochemical recognition in Australia of a rabies-like disease in a native mammal, a fruit bat, the black flying fox (Pteropus alecto). A virus with close serologic and genetic relationships to members of the Lyssavirus genus of the family Rhabdoviridae was isolated in mice from the tissue homogenates of a sick juvenile animal.


### Encephalitis Caused by a Lyssavirus in Fruit Bats in Australia

This report describes the first pathologic and immunohistochemical recognition in Australia of a rabies-like disease in a native mammal, a fruit bat, the black flying fox (*Pteropus alecto*). A virus with close serologic and genetic relationships to members of the *Lyssavirus* genus of the family *Rhabdoviridae* was isolated in mice from the tissue homogenates of a sick juvenile animal.

The *Lyssavirus* genus of the family *Rhabdoviridae* consists of five serotypes: classical rabies virus (serotype 1), Lagos bat virus (LBV) (serotype 2), Mokola virus (serotype 3), Duvenhage virus (DUVV) (serotype 4), and European bat virus (EBV) (serotype 5). The viruses within the genus share serologic relationships, but the serotypes and stable species-associated variants within serotypes can be distinguished by the reactivity profiles of monoclonal antibodies (Mab) directed against nucleoprotein and glycoprotein antigens. Analysis of the nucleotide sequence of the nucleoprotein gene has also shown genetic clusters along the same lines as serologic analysis, except that serotype 5, EBV, has been separated into two genotypes, EBV1 and EBV2 (1). Lyssaviruses have not been isolated in Australia before, although rhabdoviruses in the genus *Ephemerovirus* are present, and viruses with some serologic relationship to the *Lyssavirus* genus, for example Adelaide River virus (2), have been identified but not characterized. All members of the *Lyssavirus* genus can cause rabies or rabies-like diseases in infected animals.

Rabies-like disease has been recorded in bats on all continents except Australia. Classical rabies virus infections are common in insectivorous and hematophagous bats and less common in frugivorous bats in the Americas, while rabies-related viruses (EBV 1 and 2) are found in insectivorous bats in Europe. Two other rabies-related viruses, LBV and DUVV, are found in frugivorous bats and insectivorous bats, respectively, on the African continent. Rabies has been described in a flying fox (*Pteropus poliocephalus*) in India, although the virus causing the disease was not characterized (3). An outbreak of rabies involving several dogs occurred in the Australian island state of Tasmania in 1867 but was quickly eradicated (4). Two cases of rabies in children were reported in Australia (in 1987 and 1990). Both cases were caused by classical rabies virus and were contracted in endemic-disease countries (5). We report for the first time apparent endemic–lyssavirus-induced disease in Australia.

The four largest species of frugivorous bats in Australia are called flying foxes and belong to the genus *Pteropus* (Order Chiroptera, Suborder Megachiroptera, Family *Pteropodidae*). The Australian range of the flying foxes extends from temperate eastern and coastal Australia into the eastern tropics, around the tropical northern coastline, and down as far as the subtropical west coast. The gray-headed flying fox (*Pteropus poliocephalus*) range is the temperate and subtropical east coast, the black flying fox (*P. alecto*) inhabits primarily the subtropical and tropical range, and the little red flying fox (*P. scapulatus*) occupies the entire range except the coolest southern areas. The fourth species, the spectacled flying fox (*P. conspicillatus*) occupies a smaller range in tropical northeast Queensland. Large flying fox “camps,” with possibly tens of thousands of foxes, often contain more than one species. Analysis of population genetic markers shows a considerable movement of both *P. alecto* and *P. poliocephalus* across their geographic ranges within Australia (6). The range of *P. alecto* extends to the north of Australia into Papua New Guinea and the eastern islands of Indonesia (7,8). Regular patterns of movement suggest that flying foxes move between northern Australia and Papua New Guinea (L. Hall, pers. comm.). It is, therefore, possible that the virus described in this paper also extends across the range of these mammals outside Australia.

The flying foxes (*P. alecto*) described in this paper were wild native Australian animals collected near Ballina, in northern New South Wales, Australia. The first case, in 1996, was in a 5-month-old female black flying fox found under a fig tree, unable to fly. It was euthanized by intravenous sodium pentobarbitone injection. Fresh blood, lung, kidney, and spleen were submitted for equine morbillivirus (EMV) isolation; antibody to EMV has been detected in *P. alecto* (9), and it is conjectured that this species may be the reservoir for EMV. Paraffin-embedded formalin-fixed samples, processed by standard techniques, showed a severe nonsuppurative encephalitis. The second case, in 1995, was identified after a retrospective examination of archived paraffin-embedded tissues. The affected animal, a juvenile female of the same species, was reported to be more aggressive than usual, and was euthanized and necropsied in a similar manner to the first. Histologically, although encephalitis was very mild, many eosinophilic, cytoplasmic inclusion bodies were present in various parts of the brain. All tests for EMV were negative.

An indirect immunoperoxidase test for rabies was carried out on tissues from paraffin blocks (10) by using an antirabies Mab (HAM) (Clone 'HAM', c/o Drs. R. Zanoni and E . Peterhans, Institut für Veterinär-Virologie, Länggasstr. 122, CH-3012, Bern Switzerland) that gave good reactions without background staining when used at 1:100. The 1996 bat had positive results over wide areas of the brain, particularly in parts of the hippocampus, the mesenchymal cells of the trigeminal nucleus, and larger motor neurons of the medulla oblongata. The brain of the 1995 bat reacted strongly over all areas. The reactions were either granular, or characteristically, had ring formations in large neurons. In addition, similar reactions were seen in neuronal cytoplasms in nerve plexuses of the gastrointestinal tract from both bats. Electron microscopy examination of ultrathin sections of hippocampus from the 1996 bat showed aggregates of viral nucleocapsids within the cytoplasm of cell bodies. These inclusion bodies were specifically labeled with anti-rabies HAM Mab and gold-labeled rabbit–anti-mouse.

The only fresh samples available were blood, lung, kidney, and spleen from the 1996 bat. The blood was examined for neutralizing antibody to rabies virus (CVS-11) by the rapid fluorescent focus inhibition method (11). No neutralizing antibody was detected. Tissue homogenates (lung, kidney, and spleen) were injected into mouse neuroblastoma cells, individually injected intracerebrally into 3-week-old mice (five mice per sample), and, as a pool of the three tissues, injected into day-old suckling mice (two litters, 14 mice). No virus was isolated from cell culture after two serial passages of 4 days. One weanling mouse injected with kidney homogenate showed hind leg paraplegia 16 days postinoculation. All other mice remained normal until termination (suckling mice at 21 days and weanling mice at 28 days postinoculation). The affected mouse was euthanized, and acetone-fixed smears of brain material were positive for a lyssavirus when tested by the Centocor fluorescein-labeled Mab (Centocor Inc., 244 Great Valley Park, Malvern, PA 19355, USA). Formalin-fixed brain material showed nonsuppurative encephalitis and was positive to the indirect immunoperoxidase test for rabies virus by the HAM Mab.

Polymerase chain reaction (PCR) tests were done on nucleic acids extracted from the brain, lung, kidney and spleen of the 1996 bat and on paraffin-embedded formalin-fixed brain tissues from the 1995 and 1996 bats by using oligomers designed for the amplification of lyssavirus N protein (12) or for nested PCR amplification of the nucleocapsid protein (5). Results from these primers were consistently negative, presumably because of formalin-fixation and/or sequence heterogeneity. Therefore, another nested PCR system was devised for the amplification of N protein. Nucleic acids were extracted (5) and transcribed into cDNA by using a degenerate oligomer NP1087 (5' GAGAAAGAG[A/C]T[G/T]CAAGA[A/C/T]TA. Primary PCR was done with primers NP1087 and NP1279 (5' CAG AGACATATCT[G/C]C[G/T][G/T]ATGTG) with amplification conditions of 94°C for 1 min, 37°C for 2 min, and 72°C for 2 min for 35

Table 1. Amino acid sequences of the virus designated pteropid lyssavirus (PLV) and those of rabies and rabies-like viruses[a,b]

| | PLV[c] | PV | 4FRA | POL | 8FRA | AS | FIN | HOL | NGA | Genotype |
|---|---|---|---|---|---|---|---|---|---|---|
| PLV | - | | | | | | | | | |
| PV | 92 | - | | | | | | | | 1 |
| 4FRA | 93 | 99 | - | | | | | | | 1 |
| POL | 93 | 94 | 94 | - | | | | | | 5 |
| 8FRA | 93 | 94 | 94 | 100 | - | | | | | 5 |
| AS | 89 | 90 | 91 | 95 | 95 | - | | | | 4 |
| FIN | 85 | 87 | 88 | 87 | 87 | 84 | - | | | 6 |
| HOL | 85 | 88 | 89 | 87 | 87 | 83 | 98 | - | | 6 |
| NGA | 82 | 82 | 83 | 85 | 85 | 85 | 76 | 77 | - | 2 |
| MOK | 76 | 75 | 76 | 79 | 79 | 80 | 72 | 71 | 86 | 3 |

[a] Described in Ref. 13.

[b] Comparisons were made of cognate regions of the N protein (amino acids 298 to 426 inclusive). GenBank accession numbers are given in brackets. PV, PV rabies virus (X03673); 4FRA, fox rabies virus (U22844); POL, European bat virus (U22844, 8615POL, EBV1); 8FRA, European bat virus (U22845, 8918FRA, EBV1); AS, Duvenhage virus (U22848); FIN, European bat virus (U22846, 9007FIN, EBV2); HOL, European bat virus (U22847, 9018HOL, EBV2); NGA, Lagos bat virus (U22842), Mokola virus (U22843).

[c]PLV, the virus now reported, provisionally designated pteropid lyssavirus; PV, PV rabies virus (X03673); 4FRA, fox rabies virus (U22844); POL, European bat virus (U22844, 8615POL, ELB1); 8FRA, European bat virus (U22845, 8918FRA, EBV1); AS, Duvenhage virus (U22848); Fin, European bat virus (U22846, 9007FIN, EBV2); HOL, European bat virus (U22847, 9018HOL, EBV2); NGA, Lagos bat virus (U22842), Mokola virus (U22843).

cycles. Nested PCR was done by using primers NP1087 and NP1227 (5' CTTCA [C/T]C[G/T]ACC[A/T][C/T][C/T]GTTC ATCAT) as above except that the number of cycles was reduced to 25. PCR products were excised and sequenced. Positive PCR amplification signals were derived from the tissue culture virus and paraffin-embedded formalin-fixed brain tissues by using primers NP1087 and NP1227. Sequence analysis of these products showed that they were identifical. Sequence comparisons were done by using the nucleocapsid proteins of known lyssaviruses and the virus reported in this paper, designated pteropid lyssavirus (PLV) (Table 1). Nucleotide sequence comparisons showed that PLV had a 75% homology with LBV, 75% homology with EBV-2, and 79% with Pasteur vaccine rabies virus; at the amino acid level, the virus was 85% homologous with both EBV-2 and LBV (but 92% homologous with the rabies virus), 89% with DUVV, and 93% homologous with EBV-1 viruses. Phylogenetic analysis of both the nucleotide and amino acid sequences (not shown) showed that the virus is closely related to the EBV as well as the classic street rabies strains (12).

Brain material from the affected mouse was repassaged by intracerebral inoculation into 3-week-old mice, in which neurologic signs developed 8 to 11 days postinoculation. Examination of brain homogenate from these mice by negative-contrast electron microscopy showed classical bullet-shaped rhabdoviruses. The isolate was also passaged to mouse neuroblastoma cells, which were acetone-fixed and tested by indirect immunofluorescence using a panel of Mabs against various rabies and rabies-like viruses. The CVS-11 strain of rabies was also tested for comparison. The results (Table 2) confirm that the isolate is a lyssavirus but is different from previously described isolates. Additional nucleocapsid Mab reaction patterns (results not shown) indicated a unique profile that shared the greatest number of positive reactions with serotype 1 rabies (CVS-11) compared with published profile data on other viruses (15). Preliminary testing of the isolate in a modified (incubated 3 days) rapid fluorescent focus inhibition neutralization assay indicated that the virus was neutralized by antisera to rabies virus (mouse anti-Evelyn-Rokitnicki-Abelseth [ERA] virus). The titer of the immune mouse serum against CVS rabies virus was 1,194, and against the bat virus, 1,640.

This is the first evidence of an endemic lyssavirus in Australia. The isolate described has been provisionally called pteropid lyssavirus. The natural history of this virus in bats in Australia needs to be investigated. Further genetic and antigenic analyses are

Table 2. Reactivity patterns of nucleocapsid monoclonal antibodies (Mab) with rabies (CVS-11) and pteropid lyssavirus (PLV)

| | | IFAT[a] | |
|---|---|---|---|
| MAb[b] | Specificity[a] | CVS-11 | PLV |
| W502-2[c] | lyssavirus | + | + |
| HAM[d] | lyssavirus | + | + |
| C15-2[e] | rabies | + | - |
| 62-143-1[f] | rabies | + | + |
| 62-3-1[f] | rabies +, EBV + | + | - |
| 62-146-3[f] | rabies +, DUVV - | + | + |
| W422[c] | Mokola +, LBV + | - | - |

[a]+ indicates a positive reaction; - indicates a negative reaction
[b]Monoclonal antibody were specificities indicated by the following sources:
[c] (14,15); [d] (10); [e] (J Smith, pers. comm.); [f] (15,16)

also needed to fully determine the relationship of the virus to existing *Lyssavirus* serogroups and genogroups and to confirm its separate identity from other as yet uncharacterized rhabdoviruses isolated in Australia. The virus has been submitted to the Rabies Laboratory at the Centers for Disease Control and Prevention, Atlanta, for further Mab profile analysis and crossprotection studies with classical rabies vaccines. Findings will result in a better understanding of the public health implications of this newly emerged lyssavirus.

### Acknowledgments

We thank Peter Young, Queensland Department of Primary Industries, for conducting EMV exclusion tests and forwarding the fresh tissues to the Australian Commonwealth Scientific and Industrial Research Organization laboratory for further testing for rabies; Megan Braun and Stuart Blacksell for assisting with many of the tests; Harvey Westbury and David Boyle for professional advice; Keith Murray for assisting in the preparation of this paper; Jean Smith, Rabies Laboratory, CDC, Atlanta, Georgia, for her advice and the reagents; and Roger Kelly, University of Queensland, for his recommendation for the 'HAM' anti-rabies monoclonal antibody, which gave such good results on formalin-fixed paraffin-embedded tissues.

**Graeme C. Fraser, * Peter T. Hooper,† Ross A. Lunt,† Allan R. Gould,† Laurence J. Gleeson,† Alex D. Hyatt,† Gail M. Russell,† and Jaqueline A. Kattenbelt†**

*NSW Department of Agriculture, Wollongbar, Australia; †CSIRO Division of Animal Health, Geelong, Australia

### References

1. Bourhy H, Kissi B, Lafon M, Sacramento D, Tordo N. Antigenic and molecular characterisation of bat rabies virus in Europe. J Clin Microbiol 1992;30:2419-26.
2. Calisher CH, Karabatsos N, Zeller H, Digoutte J-P, Tesh RB, Shope RE, et al. Antigenic relationship among rhabdoviruses from vertebrates and hematophagous arthropods. Intervirology 1989;30:241-57.
3. Brass DA. Rabies in bats: Natural history and public health implications. Ridgerfield, Connecticut: Livia Press, 1994.
4. Geering WA, Forman AJ, Nunn MJ. Exotic diseases of animals: a field guide for veterinarians. Canberra: Australian Government Printing Service, 1995.
5. McColl KA, Gould AR, Selleck PW, Hooper PT, Westbury HA, Smith JS. Polymerase chain reaction and other laboratory techniques in the diagnosis of long incubation rabies in Australia. Aust Vet J 1993;70:84-9.
6. Webb NJ, Tidemann CR. Mobility of Australian flying-foxes, Pteropus spp. (Megachiroptera): evidence from genetic variation. Proc R Soc Lond Biol Sci 1996;263:497-502.
7. Ride WDL. A guide to the native mammals of Australia. Melbourne: Oxford University Press, 1970.
8. Richards GC, Hall LS. Placental Mammals, Bats, Order: Chiroptera. In Strahan R, editor. The Australian museum complete book of Australian mammals. Sydney: Angus and Robertson, 1983.
9. Young PL, Halpin K, Selleck PW, Field H, Gravel JL, Kelly MA, et al. Serologic evidence for the presence in pteropus bats of a paramyxovirus related to equine morbillivirus. Emerging Infectious Diseases 1996;2:239-40.
10. Feiden W, Kaiser E, Gerhard L, Dahme E, Gylstorff B, Wandeler A, et al. Immunohistochemical staining of rabies virus antigen with monoclonal and polyclonal antibodies in paraffin tissue sections. Zentralbl Veterinärmed 1988;35:247-55.
11. Smith JS, Yager PA, Baer GM. A rapid tissue culture test for determining rabies neutralising antibody. In: Kaplan MM and Koprowski H, editors. Laboratory Techniques in Rabies, 3rd ed. Geneva, Switzerland: World Health Organization, 1973.
12. Bouhry H, Kissi B, Tordo N. Molecular diversity of the *Lyssavirus* genus. Virology 1993;194:70-81.
13. Kissi B, Tordo M, Bourhy H. Genetic polymorphism in the rabies virus nucleoprotein gene. Virology 1995;209:526-37.
14. Wiktor TJ, Koprowski H. Antigenic variants of rabies virus. J Exp Med 1980;152:99-111.15
15. Smith JS. Rabies virus epitopic variation: use in ecologic studies. Adv Virus Res 1989;36:215-53.
16. Fekadu M, Shaddock JH, Sanderlin DW, Smith JS. Efficacy of rabies vaccines against Duvenhage virus isolated from European house bats (*Eptesicus serotinus*), classic rabies and rabies-related viruses. Vaccine 1988;6:533-9.

### Addendum

Since this report was submitted in September, 1996, the host and geographic range of the virus have been extended. The virus has been recognized by immunohistochemical techniques in five bats in three different virus isolations. Some of these bats were from another species, (the little red flying fox [*P. scapulatus*]), and from locations as far apart as 1,700 km along the Australian east coast.

## References

[PDF_00333] Bourhy H., Kissi B., Lafon M., Sacramento D., Tordo N. (1992). Antigenic and molecular characterization of bat rabies virus in Europe.. J Clin Microbiol.

[PDF_00378] Bourhy H., Kissi B., Tordo N. (1993). Molecular diversity of the Lyssavirus genus.. Virology.

[PDF_00337] Calisher C. H., Karabatsos N., Zeller H., Digoutte J. P., Tesh R. B., Shope R. E., Travassos da Rosa A. P., St George T. D. (1989). Antigenic relationships among rhabdoviruses from vertebrates and hematophagous arthropods.. Intervirology.

[PDF_00368] Feiden W., Kaiser E., Gerhard L., Dahme E., Gylstorff B., Wandeler A., Ehrensberger F. (1988). Immunohistochemical staining of rabies virus antigen with monoclonal and polyclonal antibodies in paraffin tissue sections.. Zentralbl Veterinarmed B.

[PDF_00387] Fekadu M., Shaddock J. H., Sanderlin D. W., Smith J. S. (1988). Efficacy of rabies vaccines against Duvenhage virus isolated from European house bats (Eptesicus serotinus), classic rabies and rabies-related viruses.. Vaccine.

[PDF_00380] Kissi B., Tordo N., Bourhy H. (1995). Genetic polymorphism in the rabies virus nucleoprotein gene.. Virology.

[PDF_00348] McColl K. A., Gould A. R., Selleck P. W., Hooper P. T., Westbury H. A., Smith J. S. (1993). Polymerase chain reaction and other laboratory techniques in the diagnosis of long incubation rabies in Australia.. Aust Vet J.

[PDF_00385] Smith J. S. (1989). Rabies virus epitopic variation: use in ecologic studies.. Adv Virus Res.

[PDF_00353] Webb N. J., Tidemann C. R. (1996). Mobility of Australian flying-foxes, Pteropus spp. (Megachiroptera): evidence from genetic variation.. Proc Biol Sci.

[PDF_00383] Wiktor T. J., Koprowski H. (1980). Antigenic variants of rabies virus.. J Exp Med.

[PDF_00363] Young P. L., Halpin K., Selleck P. W., Field H., Gravel J. L., Kelly M. A., Mackenzie J. S. (1996). Serologic evidence for the presence in Pteropus bats of a paramyxovirus related to equine morbillivirus.. Emerg Infect Dis.

